# Poster

**DOI:** 10.1038/sj.bjc.6601939

**Published:** 2004-06-22


### P121

### EVALUATION OF LENTIVIRAL VECTORS FOR HUMAN PANCREATIC CANCER GENE THERAPY

G Saraga , E Costello , P Ghaneh
United Kingdom, University of Liverpool, Department of Surgery, Liverpool

**Aims: 1.** To examine the suitability of lentiviral vectors (LV), based on the equine infectious anaemia virus (EIAV) and human immunodeficiency virus type-1 (HIV-1) as a gene delivery system for pancreatic cancer gene therapy and compare their efficiency. **2.** To deliver and determine the long-term stability of CapG, a cytoskeleton regulating protein, identified in our laboratory to be differentially expressed in PDAC cells compared to normal cells. **Methods:** Vectors (Oxford Biomedica UK) were produced by co-transfection of 293T cells with 1) a packaging construct, 2) a transfer construct encoding the enhanced green fluorescence protein (EGFP) and 3) a plasmid expressing the vesicular stomatitis virus glycoprotein (VSV-G) envelope protein. Vectors were concentrated by ultracentrifugation and titered on 293T cells. Particle/infectivity ratio was estimated by viral RNA assays. The efficiency of transduction was evaluated by FACS analysis for detection of EGFP. The CapG gene was cloned into the EIAV system harbouring the Neomycin resistance gene for selection of transduced cells and overexpression was determined by immunoblotting. Growth curves were constructed to investigate possible interference of the vector (cells transduced with a control vector) with normal cell growth (non-transduced cells). **Results:** Five pancreatic cancer cell lines (Panc-1, MiaPACA, Suit-2, BxPc3 and CFPAC1) have been examined and were all successfully transduced by either HIV or/and EIAV vectors. Low MOIs of 2, 3 and 10 for both vectors resulted in transduction efficiencies ranging from 3% to 47% of EGFP+ cells. Long-term overexpression of CapG was monitored for six months. No growth differences were observed between transduced and non-transduced cells. **Conclusions:** Both EIAV and HIV-based LV vectors can efficiently transduce pancreatic cancer cells. Current work involves evaluation of the vectors for infection of primary pancreatic cancer cells and also investigation of the effects of CapG overexpression. LV vectors are able of permanent integration into the host's DNA and there has been no toxicity associated with their use. We believe that LV vectors will make useful tools in pancreatic cancer research but also offer potential for pancreatic cancer gene therapy approaches.

### P122

### IN VITRO STUDIES ON NOVEL OESTROGENIC POLYAMINE DRUG CONJUGATES

H Mackay [1], RM Phillips [2], JE Brown [3], S Carrington [1]
[1] University of Bradford, Bradford, UK, [2] Tom Connors Cancer Research Centre, Bradford, UK, [3] Kingston University, Kingston-upon-Thames, UK

Polyamines are low molecular weight compounds, which have been established to play key roles in cellular growth and proliferation. Polyamines can enter cells via the specific polyamine transport system (PTS), whose expression is found to be upregulated in rapidly growing tumours making them a popular target for antitumour drug design (Delcros et al, 2002, *J. Med. Chem.* **45**: 5098). Oestrogen receptor (ER) positive (>10fmol receptor/mg cytosolic protein) (Whittliff, 1984, *Cancer* **53**: 630) breast tumours are known to display a dependency on oestrogens to maintain cell growth. It is, thereby, our aim to develop targeted antitumour agents containing polyamines, which are selective towards cells expressing the oestrogen receptor.

A trifunctional drug conjugate was synthesised which incorporated an oestrogen ligand and an intercalating acridine moiety linked via the polyamine spermine (SPM). This compound was evaluated in chemosensitivity tests using the cultured human breast cancer cell lines MCF-7 (ER+ve) and Hs578t (ER-ve). Percentage cell survival was determined after 96h exposure, using drugs in the range 0.001-100µM, via the colourimetric MTT assay (Jabber et al 1989, *Br. J. Cancer* **60**: 523). In addition doxorubicin (DOX), currently used to treat breast cancer, was also tested for a comparison of activity.

These data indicate that the trifunctional conjugate is less potent than DOX (36.7µM against 0.25µM in MCF-7). However, the conjugate does show µM activity and shows selectivity towards the ER positive cell line (36.7µM in MCF-7 against 40µM in Hs578t), hence, making it a suitable lead compound for current ongoing investigations.

### P123

### TARGETING MULTIPLE REPAIR PATHWAYS SUBSTANTIALLY ENHANCES THE SENSITIVITY OF HUMAN OVARIAN TUMOUR CELLS TO TEMOZOLOMIDE

VA. Barvaux [1], P Lorigan [2], M. Ranson [2], A. Gillum [3], R. Brown [4], R.S. McElhinney [5], TBH. McMurry [5], G.P. Margison [1]
[1] Paterson Institute for Cancer Research, Manchester, United Kingdom, [2] Christie Hospital, Manchester, United Kingdom, [3] Genta Incorporated, Berkeley Heights, United States, [4] Beatson Laboratories, Glasgow, United Kingdom, [5] Trinity College, Dublin, Ireland

Temozolomide is an alkylating agent that mediates its cytotoxic effects *via* $O^6$-methylguanine ($O^6$-meG) adducts in DNA and their recognition and processing by the postreplication mismatch repair system (MMR). $O^6$-meG adducts can be repaired by the DNA repair protein $O^6$-alkylguanine-DNA-alkyltransferase (MGMT), which therefore constitutes a major resistance mechanism to the drug. Resistance to Temozolomide can also be mediated by loss of MMR, which is frequently mediated by methylation of the *hMLH1* gene promoter. Methylation of hMLH1 can be reversed by treatment of cells with 5-aza-2'-deoxycytidine, while the MGMT pseudosubstrate $O^6$-(4-bromothenyl)guanine (PaTrin-2; Patrin™; lomeguatrib$^{BAN}$, KuDOS) can deplete MGMT activity. Using a drug resistant cell line which expresses MGMT and has methylated *hMLH1*, we show that while either of these treatments can individually sensitize cells to Temozolomide, the combined treatment leads to substantially greater sensitization. The increased sensitization is not observed in matched MMR proficient cells. Furthermore, we show that attenuation of the anti-apoptotic protein Bcl-2 by the antisense oligonucleotide Oblimersen sodium (Genasense™, Aventis/Genta) leads to the sensitization of the MMR proficient cells. This effect is also enhanced by the combined treatment of Oblimersen and PaTrin-2.

This work is supported by the European Union Marie Curie Individual Fellowship (proposal nº 2000-02021), the Bourse post-doctorale de recherche scientifique (Mai 2000) of the Université Catholique de Louvain, Belgium, and Cancer Research UK

### P124

### IS 3, 4, 4' ,5-TETRAMETHOXYSTILBENE (DMU212) A BETTER CANCER CHEMOPREVENTIVE AGENT THAN *TRANS*-3,4', 5-TRIHYDROXYSTILBENE (RESVERATROL)?

S Sale [1], RG Tunstall [1], GA Potter [2], WP Steward [1], AJ Gescher [1]
[1] United Kingdom, Leicester University, Leicester, [2] United Kingdom, DeMontfort University, Leicester

Resveratrol is a naturally occurring polyphenol with cancer chemopreventive properties in preclinical models of carcinogenesis, including those of colorectal cancer. Recently a novel analogue of resveratrol, DMU212 has been found to exhibit preferential growth-inhibitory and pro-apoptotic properties in transformed cells, when compared with their untransformed counterparts (Lu *et al*., 2001 *Carcinogenesis,* **22***:* 321), this activity was superior to that of resveratrol. In order to aid the rational development of resveratrol and DMU212 as colorectal cancer preventive agents, $Apc^{Min/+}$ mice received these stilbenes, at three different doses (0.05, 0.2 and 0.5% admixed in the diet), from weanling age to 18 weeks. At 18 weeks adenoma numbers were scored. Resveratrol at all doses reduced adenoma number to 96.7 ± 31.1, 73.2 ± 9.6, and 69.7 ± 12.7 per cent of control, respectively, the two higher doses being significantly different from control ($p<0.05$). DMU212 at the same doses reduced adenoma number to 85.0 ± 16.0, 76.1 ± 8.2 and 88.1 ± 21.9 per cent of the control, respectively. The difference was significant only in the case of the 0.2% dose ($p<0.05$). COX-2, which is thought to contribute to cancer progression, was measured by Western blotting in HCA-7 colon cancer cells after incubation with stilbenes for 24, 48 and 96 hr. Resveratrol showed significant inhibitory effects on COX-2 expression at 48hr and 96 hrs at 10µM ($p<0.05$), whilst DMU212 had a minimal effect. Prostaglandin $E_2$ ($PGE_2$), a product of COX enzyme activity and an indirect indicator of COX-2 activity, was also investigated. Resveratrol and DMU212 showed similar effects on $PGE_2$ levels, with 10 and 50µM being the most potent, causing up to 96% reduction. These findings provide a clue as to the mode of action of these compounds. These results provide a strong rationale to further investigate resveratrol and DMU212 as possible colorectal cancer chemopreventive agents.

© 2004 Cancer Research UK

### P125

**ANTI-METASTATIC EFFECTS OF THE BISPHOSPHONATE ESTER APOMINE IN BREAST CANCER CELL LINES *IN VITRO***

LM Pickering , LC Lowe , SJ Ganguli , JL Mansi , KW Colston
St. George's Hospital Medical School, London, United Kingdom

Apomine is a novel 1,1-bisphosphonate ester that inhibits cholesterol synthesis through mevalonate pathway inhibition and mimics the effects of farnesol *in vitro*. Unlike conventional bisphosphonates, it does not localise exclusively to bone and has a plasma half-life of 156 hours. There is little data addressing the anti-neoplastic effects of apomine but growth inhibitory and apoptotic effects have been observed in a number of cancer cell types, mediated through activation of the farnesoid X receptor (FXR), and we have described apoptosis induction in breast cancer cells *in vitro*. Breast cancer metastasises to bone in one third of cases and adhesion of cancer cells to bone matrix is one critical step in this process. This study therefore investigated the consequences of apomine treatment for breast cancer cell adhesion.
Breast cancer cell lines (n=2) were exposed to apomine for 24 - 72 hours. Equal numbers of viable cells were seeded onto mineralised dentine and unmineralised protein matrices for 1-24 hours. Adherent cells were washed, fixed, stained and the dye eluted or cells counted. Cell growth and viability were assessed by Sulforhodamine B and MTS dye reduction assay.
Breast cancer cells adhered to all matrices under control conditions. Exposure to apomine for 24 hours at concentrations of 1 and 10 microM was associated with 79 and 83% reduction in adhesion, 26 and 52% inhibition of proliferation but insignificant reduction in viability. Prolonged treatment with 10 microM apomine resulted in more pronounced reduction in proliferation and viability by up to 73 and 54% respectively after 3 days of treatment.
This study confirms that apomine has growth inhibitory properties in breast cancer cells and provides the first demonstration that it also inhibits their adhesion. Future studies will determine whether this effect is mediated by FXR activation or mevalonate pathway inhibition. These findings indicate that apomine has clinical anti-metastatic potential.

### P126

**DEVELOPMENT OF A REAL-TIME RT-PCR ASSAY FOR THE QUANTIFICATION OF DRUG-METABOLISING ENZYMES AND TARGET OF CAPECITABINE**

JS Macpherson [1], S Guichard [2], DI Jodrell [2]
[1] Cancer Research UK, Edinburgh, United Kingdom, [2] University of Edinburgh, Edinburgh, United Kingdom

Capecitabine is a new fluoropyrimidine, prodrug of 5-fluorouracil (5FU) approved for the chemotherapeutic treatment of breast and colon cancers. Capecitabine is metabolised in both liver and tumour tissues by carboxylesterases (hCES), cytidine deaminase (CDD) and thymidine phosphorylase (TP) to form 5FU, which is inactivated by dihydropyrimidine dehydrogenase (DPD) reducing its capacity for interaction with its target, thymidylate synthase (TS). These enzymes have different distributions in liver and tumour tissue and may impact on the activation/inactivation of the drug. We have developed a real-time PCR assay to determine the expression of the 2 isoforms of carboxylesterase (hCES1 and hCES2), CDD, TP, DPD and TS in a panel of 6 colon cancer cell lines. RT-PCR was performed on a Rotorgene 3000 (Corbett Research). Calibration curves were made from HCT-8 RNA (0.05-50 ng) and results expressed as equivalent ng of HCT-8 RNA. The precision of the method was evaluated within-runs and in-between runs on 2 different batches of RNA. Three out of 6 cell lines lack CES1 expression and SW620 had a very low level (0.05±0.02 eq ng HCT-8 RNA). CES2 expression varied from 0.38±0.08 in HCT-116 to 2.6±0.1 in SW620. CDD expression ranged from 0.3±0.03 in SW620 cells to 4.9±0.7 in COLO205. HT29 and HCT-116 had very high levels of TP (18±4 and 15±6, respectively). Variability between replicates within the same run was low and variability of 18S between subsequent runs within the same RNA batch ranged from 0.01% for SW620 to 20% for HT-29. Variability between batches was greater varying from 7-22% for CES1, to 13-68% for CDD. The use of 18S to normalize the results decreased only the variability of CDD expression. The real-time PCR method developed is reproducible and the variability between batches could be linked to the confluency of the cells used for RNA preparation. This method could also be used in patients treated with the drug.

### P127

**PROTEIN PRENYL TRANSFERASE INHIBITION IN PROSTATE CANCER USING AZD3409**

R. Khafagy [1], T.C. Stephens [2], C. Hart [1], V. Ramani [1], M.D. Brown [1], N.W. Clarke [3]
[1] The Paterson Institute, Christie Hospital NHS Trust, Manchester, United Kingdom, [2] AstraZeneca, Alderley Park, Maccelsfield, Cheshire, United Kingdom, [3] Hope Hospital, Salford, United Kingdom

Prostate cancer is the most common malignancy in men with a predilection to metastasise to the bone. There is a need for a chemotherapeutic agent capable of preventing or slowing down the growth of bone metastases. AZD3409 is a novel protein prenyl transferase inhibitor with anticancer potential. **Methods:** The effect of escalating doses of AZD3409 on proliferation of the malignant PC-3 and LNCaP, and non-malignant PNT2-C2 prostate epithelial cell lines was determined by CASY TT cell counter. PC-3 and PNT2-C2 colony growth and formation was studied in co-culture using long-term human bone marrow stroma harvested from individuals with benign disease using standardized quantitative techniques in the presence of escalating doses of AZD3409. Prostatic cellular invasion through matrigel (synthetic basement membrane) and cultured endothelial monolayers was measured in invasion chambers in the presence and absence of drug using cytokeratin labelled quantitation of migrating cells. **Results:** AZD3409 displayed marked anti-proliferative properties on all the non-malignant (PNT2-C2) and malignant (PC-3 and LNCaP) prostate epithelial cells ($IC_{50}$=10 nM after 5 days exposure). AZD3409 inhibited invasion through bone marrow endothelium towards bone marrow stroma, with a concentration of 1000μM inhibiting 50% of PC-3 invasion. Pre-treatment resulted in a 1000-fold decrease in the invasion $IC_{50}$ with inhibition of invasion occurring at 1 nM, levels known to be attainable *in vivo*. AZD3409 inhibited colony formation in bone marrow stroma co-cultures resulting in reduced numbers of colonies (50% inhibition at 4 μM) and a reduction in colony size (50% reduction at 8 μM). **Conclusion:** AZD3409 is a potent inhibitor of prostate epithelial cell proliferation and its anti-colony formation in bone marrow stroma co-culture suggests potential as an anti-metastatic agent. Moreover, AZD3409 shows significant inhibition of prostate cancer cell invasion through human bone marrow endothelium.

### P128

**ACTIVITY OF MDI-301, A NOVEL SYNTHETIC RETINOID, AGAINST BREAST CANCER IN VIVO**

MVCL Appleyard [1], MA O'Neill [1], KE Murray [1], SE Bray [1], GA Thomson [1], W Purcell [2], AM Thompson [1]
[1] Ninewells Hospital - University of Dundee, Dundee, United Kingdom, [2] Molecular Design International, Inc(MDI), Memphis, United States

Natural and synthetic retinoids are known to elicit antiproliferative actions in many cancer cells including breast, gastric and ovarian cancers, and neuroblastoma. However, the erythema and skin irritation induced by such compounds limit their use as therapeutic agents. MDI-301 is a synthetic retinoid in which an ester linkage replaces the carboxylic acid of 9-cis retinoic acid. It has recently been shown that MDI-301 stimulates epidermal and dermal thickening following systemic or topical application with no irritation to the skin of hairless mice**.** The efficacy of MDI-301 was investigated against established MCF7 (ER-positive, high expression of Retinoic Acid Receptor-Alpha) and MDA-MB-231 (ER-negative, low expression of Retinoic Acid Receptor-Alpha) breast cancer xenografts.
**METHODS**: Nunu mice where injected subcutaneously with MCF7 or MDA-MB-231 cells. After tumour development mice were divided into 3 groups: 1: untreated; 2: vehicle control (DMSO + PBS) and 3: MDI-301 in DMSO + PBS at 10 mg/kg for 14 days by the intraperitoneal route.
**RESULTS**: Mice bearing MCF7 tumours and receiving vehicle alone had a mean increase in tumour volume of 500% (± 116) after 14 days and a doubling time of 6 days; mice treated with MDI-301 exhibited a mean increase of 260% (±100) in tumour volume after 14 days and a doubling time of 9 days. This corresponds to a significant reduction in tumour growth of 48% (Student's t-test $p < 0.05$). MDI-301 showed no antiproliferative effect against MDA-MB-231 xenografts. There was no weight-loss or signs of toxicity in the treated group.
**CONCLUSION:** The efficacy of MDI-301 in inhibiting *in vivo* the growth of MCF7 tumours without evidence of irritation or toxicity makes this drug a novel candidate for breast cancer therapy.

© 2004 Cancer Research UK

### P129

### BISPHOSPHONATES INDUCE APOPTOSIS AND INHIBIT ADHESION TO MINERALISED BONE MATRIX IN HUMAN LUNG CARCINOMA CELL LINES

CD Macdonald, LM Pickering, JL Mansi, KW Colston
St. George's Hospital Medical School, London, United Kingdom

Lung carcinoma frequently metastasises to bone causing a substantial clinical problem. Bisphosphonates (BPs) limit the progression of bone metastases in several cancers, including lung cancer. The mechanisms involved are not fully understood but BPs are known to have direct effects in cancer cells *in vitro*, including apoptosis induction and inhibition of invasive properties. Little data exist addressing the effects of BPs in lung cancer cells; we therefore investigated the consequences of BP treatment for lung cancer cell survival and adhesion.
Two nitrogen-containing BPs zoledronic acid (ZOL) and pamidronate (APD) and the non nitrogen-containing compound clodronate (CLOD) were tested against lung cancer cell lines (n=3). Cell viability and number were determined by MTS dye reduction and Sulforhodamine B assay. Apoptosis was assessed by Cell Death ELISA. Cleavage of Ac-DEVD-pNA was used to determine caspase activation. Inhibition experiments were performed with the caspase inhibitor Z-VAD-FMK. The effects of ZOL on adhesion were assessed by seeding equal numbers of viable cells onto dentine matrix for 24 hours. Adherent cells were washed, fixed, stained and counted.
BPs reduced cell viability and number in a concentration and time dependent manner. ZOL and APD were consistently more potent than CLOD. All BPs induced apoptosis by day three of treatment and this was associated with caspase activation to varying degrees. BP-induced loss of cell viability was partially abrogated by co-incubation with Z-VAD-FMK. Exposure to 1 or 100 microM ZOL for 24 hours led to a substantial reduction in adhesion to mineralised dentine, whereas 100 microM EDTA had minimal effect.
This study demonstrates that BPs directly induce apoptosis in human lung carcinoma cells by both caspase dependent and independent means and inhibit their adhesion to mineralised dentine. These observations provide a scientific basis for the clinical effects of BPs in lung cancer.

### P130

### HSABODIES: A NEW CLASS OF ENGINEERED ANTIBODY-BASED MOLECULES FOR TARGETING CANCER

A Huhalov [1], C Graff [2], KD Wittrup [2], B Tolner [1], Pedley RB [1], W Vervecken [3], R Contreras [3], RHJ Begent [1], KA Chester [1]
[1] United Kingdom, Royal Free & University College Medical School, London, [2] United Kingdom, Massachusetts Institute of Technology, Cambridge, [3] United States, Ghent University, Gent-Zwijnaarde

It was hypothesised that a human antibody-based molecule which had a long residence time in tumour and a controllable systemic clearance would be a superior cancer targeting agent in terms of immunogenicity, tumour uptake and tumour:normal tissue ratios. To test this, a novel fusion protein was engineered incorporating human serum albumin (HSA) as a backbone to genetically link two high affinity, humanised derivatives of the anti-carcinoembryonic antigen (CEA) single chain Fv antibody fragment MFE-23. Asn-linked glycosylation sites were engineered into the HSA linker to modulate its pharmacokinetics by directing clearance through mannose receptors. The divalent MFE-23-HSA assemblies, termed HSAbodies, were expressed in *Pichia pastoris* by fermentation and purified using immobilised metal- and CEA-affinity chromatography. BIACore analysis indicated that the purified proteins were functionally divalent in terms of CEA binding.
*In vivo* studies demonstrated that the $^{125}$Iodine-radiolabelled HSAbodies specifically localised to LS174T human colon carcinoma xenografts whilst clearing rapidly from normal tissues. Overall tumour uptake for the unglycosylated HSAbody reached maximum levels of 24.5±12.5% injected dose (ID)/g tissue at 24 h following injection, with a corresponding tumour: blood ratio of 7:1. The presence of mannose glycosylation on the HSAbody resulted in its accelerated systemic clearance compared to the unmodified protein, with subsequently less overall uptake in the tumour (3.5±2.5% ID/g at 24 h) but a substantial increase in tumour: blood ratios, with levels reaching up to 22:1 at 24 h and as high as 92:1 at 48 h.
A novel class of CEA-targeting molecules has been described which combines molecular design features such as humanisation, high affinity and avidity to achieve long tumour retention and optional circulating half-life. These features indicate a clear therapeutic and diagnostic potential for HSAbodies in both native and glycosylated form.

### P130:1

### ENHANCED *IN VIVO* SELECTION OF RETROVIRALLY TRANSDUCED HAEMOPOIETIC STEM CELLS USING HOXB4 AND MGMT

MD Milsom, LB Woolford, D Gagen, LJ Fairbairn
United Kingdom, Paterson Institute for Cancer Research, Manchester

Recent clinical trials in haemopoietic cell gene therapy have highlighted the importance of an *in vivo* selective advantage in achieving therapeutic levels of gene-modified cells. We have developed a tri-cistronic vector that co-expresses HoxB4 with the drug-resistance factor MGMT and eGFP. Using an *in vivo* competitive repolulation/selection assay, we have demonstrated that this vector confers a profound engraftment advantage on transduced haemopoietic stem cells, which is further amplified following application of an *in vivo* selection pressure using an $O^6$-alkylating agent. However, we have also shown that over-expression of HoxB4 in a multi-potent haemopoietic cell line leads to a delay in myeloid and erythroid differentiation – a potential step towards transformation. To reduce this risk, we have developed a drug-inducible form of HoxB4 who's activity is dependent on tamoxifen and have shown that this confers a tamoxifen-dependent advantage to haemopietic stem cells both *in vitro* and *in vivo*. This combination of a powerful, inducible selective advantage with efficient expression via tri-cistronic vectors may allow the development of safer and more effective gene therapy strategies.

### P130:2

### SYNTHESIS AND ANALYSIS OF SPIRUCHOSTATIN A, A POTENT BICYCLIC TETRAPEPTIDE HISTONE DEACETYLASE INHIBITOR

G Packham [1], A Yurek-George [2], F Habens [1], M Brimmell [1], A Ganesan[2]
[1] United Kingdom, Cancer Research UK Oncology Unit, University of Southampton School of Medicine, Southampton, [2] United Kingdom, School of Chemistry, University of Southampton, Southampton

There is considerable excitement that histone deacetylase inhibitors (HDACi) may be a very important new class of anti-cancer drugs, inducing growth arrest and apoptosis in a wide range of cancer cells with relatively little effect on normal cells. FK-228, a bicyclic tetrapeptide with potent HDACi activity, has produced encouraging results in clinical trials. FK-228 functions as a natural pro-drug and unlike most other HDACi, possesses some selectivity versus individual HDAC enzymes. Therefore, the further development of this class of compounds is of major importance. However, since FK-228 is a natural product isolated by fermentation, it is unlikely to be optimal for inhibiting human HDACs and has not been amenable for structural modification. We have now developed a novel synthetic route for Spiruchostatin A, a recently isolated natural product resembling FK-228. We have demonstrated that Spiruchostatin A is a bona fide HDACi since, similar to trichostatin A, it induced accumulation of acetylated histones and activated the p21$^{cip1}$ promoter (a well studied HDACi-inducible promoter) in MCF7 breast cancer cells. Moreover, Spiruchostatin A inhibited the growth of MCF7 cells at approx. ten-fold lower concentrations than TSA ($IC_{50}$ for growth inhibition of 10 nM and 100 nM for Spiruchostatin A and TSA, respectively). We also synthesised an analogue of Spiruchostatin A, epimeric at the beta-hydroxy acid fragment. By altering the stereochemistry at this position, the potential interactions of the tetrapeptide "cap" of Spiruchostatin A with amino-acids surrounding the active site of the target enzyme would be radically altered. Consistent with the idea that the "cap" structure is an important determinant for HDAC binding, epi-Spiruchostatin A did not inhibit the growth of cancer cells and does not activate the p21 promoter.

© 2004 Cancer Research UK

### P130:3

### OLIGOSACCHARIDE DERIVATIVES OF LOW-MOLECULAR WEIGHT HEPARIN AS ANGIOGENESIS INHIBITORS

J Hasan [1], S.D Shnyder [2], M Bibby [2], A Clamp [1], R Byers [3], G.C. Jayson [1]
[1] United Kingdom, Paterson Institute for cancer research/ Christie Hospital, Manchester, [2] United Kingdom, Cancer research unit,Univerist of bradford, Bradford, [3] United Kingdom, University of Manchester, Manchester

The aim of this study was to define the anti-angiogenic activity of length-defined oligosaccharide derivatives of low-molecular weight heparin in a novel in vivo model of angiogenesis, the hollow fibre assay. Hollow fibres are made of polyvinylidene fluoride with an internal diameter of 1mm and a molecular weight cutoff of 500,000 Da. This allows pro-angiogenic growth factors produced by cells within the fibres to migrate and stimulate angiogenesis in surrounding tissues whilst cells themselves are retained within the fibre. Heparan sulfate oligosaccharides were obtained by partial depolymerisation of Innohep (tinzaparin sodium) using heparinase I. Oligosaccharides ranging in size from hexasaccharides to tetradecasaccharides (dp 6-dp14) were used. Hollow fibres measuring 1.5cm in length were set up using freshly harvested H460 non-small cell lung carcinoma cells (NSCLC) at a seeding density of 2.5 x $10^5$cell/ml. This cell line elaborates a host of pro-angiogenic growth factors, principally VEGF. Under light inhalation anaesthesia, one hollow fibre was implanted subcutaneously in the lower left dorsal area and one fibre in the lower right dorsal area of male B+K nude mice using the small tumour implant trocar (day0). Oligosaccharides (20mg/kg) were administered intraperitoneally between day 1 and day 23. The animals were sacrificed and fibres harvested on the day following the last injection. The treatment groups were as follows (5 animals per group):
Dp6 (20mg/kg/day)
Dp 8 (20mg/kg/day)
Dp 10 (20mg/kg/day)
Dp12 (20mg/kg/day)
Dp 14 (20mg/kg/day)
Untreated control groups: (a) fibres + cells (Positive control)
(b) Fibres + medium only (Negative control)
An MTT assay was performed on one fibre per animal to confirm adequate cell viability. Paraffin-embedded sections were stained with a pan-endothelial marker (von Willebrand factor) and neoangiogenesis quantified by manual microvessel counting (x400 magnification). Microvessels were counted in the new granulation tissue that envelopes the hollow fibre. Microvessel counts were performed by a single observer in a blinded fashion to minimise bias. Of the oligosaccharides tested (dp6-dp14), the octasaccharides (dp8s) significantly inhibited angiogenesis compared to the untreated control (point estimate 0.56, 95%CI 0.34-0.94).
Conclusion: The shorter length oligosaccharide derivatives of Tinzaparin, principally octasaccharides, inhibit the pro-angiogenic activity of a H460 NSCLC cell line in vivo and could be potentially developed as anti-angiogenic agents.

### P130:4

### OSTEOPONTIN (OPN) IS DIFFERENTIALLY ACTIVATED BY TWO LOW-CALCEMIC ANALOGS OF 1α,25-DIHYDROXYVITAMIN D3 (VD3) BUT INHIBITED BY TCF-4.

M McCann [1], M El-Tanani [1], G Posner [2], F.C Campbell [1]
[1] United Kingdom, Surgery, Cancer Centre, The Queen’s University of Belfast, Belfast, [2] United Kingdom, Dept of Chemistry, Faculty of Arts and Sciences, Johns Hopkins, Baltimore

**Introduction:** Osteopontin (OPN) is implicated in colorectal cancer invasion and metastasis. Regulation of OPN transcription may be important for CRC prevention. Ligand activated nuclear vitamin D receptor (nVDR) binds to the response element (VDRE) of the OPN promoter and may have indirect effects on OPN transcription through the β-catenin/Tcf-4/Lef-1 pathway.
**Aim:** This study tests the hypotheses that (i) low-calcemic analogs of vitamin D3 activate OPN transcription in proportion to their VDR-mediated transcriptional potencies and (ii) components of the β-catenin/Tcf-4/Lef-1 pathway influence ligand-nVDR activated OPN transcription.
**Methods:** Rama 37 cells were transiently transfected with an OPN promoter-luciferase reporter construct with or without human nuclear VDR (hnVDR) cDNA, before treatment with either VD3, high activity (QW) or low activity (BTW) low-calcemic VD3 analogs, across a dose range. Cells were then co-transfected with β-catenin, Tcf-4, Lef-1 or a combination of their cDNAs and the effects on OPN transcription were assessed.
**Results:**OPN transcription was induced by >50 fold by QW $10^{-9}$M vs BTW $10^{-7}$M (vs VD3 [$10^{-8}$M]). Transfection of cells with β-catenin and Lef-1 cDNAs, enhanced OPN transcription. Conversely, Tcf-4 strongly inhibited spontaneous or VD3 stimulated OPN transcription (eg OPN + hnVDR + VD3 $10^{-8}$M vs OPN + hnVDR + VD3 $10^{-8}$M +Tcf-4 =110 vs 24.7 RFU’s).
**Conclusions:** Low-calcemic analogs QW and BTW activate OPN transcription in proportion to their VDR-mediated transcriptional activities. Tcf-4, a component of the nVDR responsive β-catenin/Tcf-4/Lef-1 pathway, inhibits ligand-nVDR activated OPN transcription. These molecular mechanisms may provide novel therapeutic targets for CRC prevention.

### P130:5

### CYCLOPHOSPHAMIDE INDUCED CD11B$^+$ CELLS CAN INHIBIT ANTI-CD40 MONOCLONAL ANTIBODY THERAPY OF B-CELL LYMPHOMA

J Honeychurch , M J Glennie , T M Illidge
United Kingdom, Cancer Sciences Division, Southampton University, Southampton

Immunotherapy is emerging as a promising strategy for the treatment of B-cell lymphoma. Some immunomodulatory monoclonal antibodies (mAb) such as anti-CD40, have shown significant activity in pre-clinical models. Evidence is emerging to suggest that by combining these mAb with conventional treatments, such as chemotherapy, the anti-tumour effects may be further enhanced. Here, we have assessed the efficacy of combining anti-CD40 mAb with the commonly used cytotoxic drug cyclophosphamide (CY).
Initially, we conducted a number of immunotherapies against the well established $BCL_1$ lymphoma model. As anti-CD40 mAb can be therapeutic alone, we used sub-optimal doses of the mAb to look for possible synergistic effects. Interestingly, timing of CY relative to mAb appeared critical to therapeutic outcome. Cohorts given CY 7 days prior to anti-CD40 (d7 CY) show reduced survival compared to those receiving mAb alone, whereas treatment with CY 1 day before, or on the same day as mAb results in increased survival. In vivo tracking experiments revealed that around 5 days after anti-CD40 treatment there is a potent CTL response that clears tumour. However, d7 CY therapy results in reduced CTL expansion but an increased number of splenic CD11b$^+$ cells compared to other treatments. These CD11b$^+$ cells have an immature myeloid precursor phenotype and are able to produce nitric oxide (NO). Co-culture of CD11b$^+$ cells with murine splenocytes results in greatly reduced CTL proliferation, but not apoptosis, that can be reversed in the presence of NO inhibitors. Similarly, splenic CTL proliferation in vivo is inhibited by this increased population of CD11b$^+$ cells and adoptive transfer of purified CY induced CD11b$^+$ in to non-CY treated mice inhibits anti-CD40 therapy of $BCL_1$.
This data indicates that combining CY with immunotherapy can enhance therapeutic responses, but the timing of CY relative to immunotherapy is critical to therapeutic outcome.

### P130:6

### DETERMINATION OF ONDANSETRON IN PLASMA AND ITS PHARMACOKINETICS FROM CANCER PATIENTS AFTER CHEMOTHERAPY

J Yu , C.J Ward , S Brown , C.R. Wolf , E.M. Rankin
United Kingdom, University of dundee, Dundee

Ondansetron is an antiemetic widely prescribed to patients undergoing chemotherapy. Side effects include constipation, nausea, vomiting and headache. As partly ongoing study into inter-individual variability in pharmacokinetics from cancer patients, we developed a high performance liquid chromatography-tandem electrospray mass spectrometric assay for quantification of ondansetron in plasma from cancer patients.undergoing chemotherapy. The liquid chromatographic separation was achieved on a phenyl column with a gradient mobile phase of aqueous 20 mM ammonium acetate-acetonitrile at a flow rate of 0.2 ml/min. The assay was validated between 1.0 to 200 ng/ml with tropisetron as the internal standard. The intra- and inter-day precision did not exceed 12.5% and 11.1% respectively. The intra- and inter-day accuracy did not exceed 16.5% and 6.8% at five different quality control samples (1.0, 2.5, 25, 150 and 200ng/ml). The Application of the assay to determine pharmacokinetics of ondansetron in cancer patients receiving chemotherapy is described.

© 2004 Cancer Research UK

### P131

### ANALYSIS OF THE ROLE OF THE MDM2-P53 PATHWAY IN TUMOUR EVOLUTION IN RENAL CELL CARCINOMA

HE Warburton [1], KF Parsons [1], MW Linehan [2], MT Boyd [1]
[1] United Kingdom, University of Liverpool, Department of Surgery, Liverpool, [2] United Kingdom, National Cancer Institute, Urologic Oncology Branch, Bethesda

A major obstacle to the management of renal cell carcinoma (RCC) is the paucity of reliable prognostic indicators. Two lines of evidence support the conclusion that the MDM2-p53 pathway might provide such an indicator. Firstly a survey of 120 cases of RCC found that high levels of p53 and MDM2 frequently occur in renal cell carcinoma (19% of patients) and that this phenotype has the poorest prognosis with a mean disease free survival time of 0.8 years c.f. >5 years in patients with low or undetectable p53 and MDM2. Secondly, MDM2 is located on 12q13 and gain of 12q has been found to be one of the strongest predictors of poor prognosis.
We examined the level of MDM2 protein, RNA and DNA in a panel of 11 RCC cell lines. We have found that 5/11 of the RCC cell lines express higher levels of MDM2 protein than U2OS osteosarcoma cells. This occurs in the absence of MDM2 amplification or transcriptional up-regulation. It has previously been found that MDM2 protein can be up-regulated by a process of enhanced translation and we are investigating this and also the stability of the MDM2 protein.
Given the pattern of mutations described above, it is unlikely that MDM2 up-regulation arises prior to p53 mutation/LOH. In cell lines, over-expression of MDM2 is not generally achievable by stable transfection, because of ill-defined toxicity associated with MDM2 expression. Thus we suggest, that in RCC (similar to bladder cancer) acquisition of MDM2 up-regulation confers an advantage to RCC cells promoting an aggressive phenotype but this only occurs in p53 mutant cells. We have introduced both MDM2 and a dominant negative p53 mutant into RCC cells that possess low levels of MDM2 protein and wt p53 either: MDM2 first, p53 second or vice versa to mimic apparent tumour evolution. We have examined the cells' viability and proliferation to determine whether RCC cells can tolerate high levels of MDM2 only in the presence of mutant p53.

### P132

### A POTENTIAL ROLE FOR THE E7 PROTEIN OF HIGH RISK HUMAN PAPILLOMAVIRUSES IN IMMUNE EVASION

G Bottley , GE Blair
United Kingdom, University of Leeds, Leeds

Many human viruses encode proteins that enable infected cells to evade the actions of the cellular immune system. Similarly, most human tumour cells show down-regulation of the major histocompatibility complex (MHC) class I pathway of antigen processing and presentation, thus resulting in an escape from immunosurveillance by cytotoxic T cells. Human Papillomaviruses (HPV) of the high risk group (such as HPV 16,18 and 31) cause a range of ano-genital cancers (for example cervical cancer) and are the most significant virally-induced human cancers.
We have been investigating the role of the E7 protein from the high risk HPV in antigen processing and presentation. Previous studies have suggested that expression of E7 results in transcriptional down-regulation of MHC class I. However, this needs to be extended by analysing any functional change in antigen processing that is directly attributable to the E7 protein. It has proved difficult to examine the role of E7 alone, as E7 has pro-apoptotic activity, normally countered by the viral E6 protein.
Recent advances have allowed two new approaches to this problem. Inhibition of E7 translation with specific small interfering RNA (siRNA) is able to "knock out" E7 expression in HPV 16-transformed cell lines and it is then possible to examine antigen processing in these cells. Inhibition of E7 expression has confirmed that cells enter a replicative senescent state, with an associated $G_1$ arrest. These cells also appear to subtly modulate their antigen processing pathways, although it is not yet clear whether this is a direct effect of loss of E7 or linked to the senescent state of the cells. Another approach that we have taken is the use of cell lines stably transfected with a plasmid construct in which an inducible promoter is linked to the HPV 16 E7 gene. Removal of Tetracycline from the culture medium results in expression of E7. Current studies are directed towards analysis of MHC class I expression in both experimental systems.

### P133

### SIRNA-MEDIATED DEPLETION OF FGFR1 INHIBITS BFGF-INDUCED APOPTOSIS IN CELLS OF THE EWING'S SARCOMA FAMILY OF TUMOURS (ESFT)

JR Boyne , SA Burchill, Candlelighter's Research Laboratory, Leeds

Treatment of the ESFT cell line TC-32 with basic fibroblast growth factor (bFGF) leads to cell death through a caspase-8 and 3 dependent mechanism which is accompanied by an up-regulation of $p75^{NTR}$ (Westwood et al, 2002, Oncogene, 21(5): 809-824). In this study we have investigated the role played by FGFR1 signalling in the initiation of bFGF-induced cell death.

Viable cell number was quantified using the trypan blue exclusion assay and apoptosis by fluorescent associated cell sorting (FACS) of annexin V/PI labelled cells. Total and phosphorylated FGFR1, ERK and $p38^{MAPK}$ were analysed by Western blot. FGFR phosphorylation was inhibited using the small molecule SU5402 (Merck Biosciences), and FGFR1 expression depleted using siRNA.

bFGF (20ng/ml) induced phosphorylation of FGFR1 in TC-32 cells after 2 minutes exposure. This was accompanied by a sustained activation of Ras/ERK and $p38^{MAPK}$. Pre-treatment of TC-32 cells with SU5402 (20μM) rescued 92% of cells from bFGF-induced cell death, consistent with the hypothesis that bFGF-induced cell death is mediated through an FGFR dependent pathway. SU5402 inhibited phosphorylation of ERK/$p38^{MAPK}$ and the up-regulation of $p75^{NTR}$.

FGFR1 was depleted at the mRNA level using siRNA. Specific depletion of FGFR1 was confirmed by real-time PCR. Western blot analysis demonstrated that FGFR1 was depleted at the protein level compared to a scramble siRNA control. Depletion of FGFR1 in TC-32 cells prevented up-regulation of $p75^{NTR}$ and inhibited bFGF-induced cell death (100%).

In summary, bFGF-induced cell death in the ESFT cell line TC-32 requires functional FGFR1 signalling.

### P134

### DEFINING POTENTIAL TARGETS FOR CELL-BASED IMMUNOTHERAPY OF COLORECTAL CANCER

SE Perry , GP Cook , AA Melcher , P Quirke , GE Blair
University of Leeds, Leeds, United Kingdom

Despite significant advances in surgery, radiotherapy and chemotherapy, cure rates for colorectal cancer (CRC) remain low and novel approaches to treatment are urgently needed. Immunotherapy holds great promise for cancer therapy, if suitable targets can be identified. We are therefore exploiting the results of microarray analysis to identify target proteins that are significantly up-regulated in CRC and contain T cell epitopes. We are currently evaluating several proteins for their potential in cell based immunotherapy. Here we describe data on one such protein, CDCP1 (a Cub Domain Containing Protein), a cell-surface molecule identified by its elevated expression in colorectal cancer tissues. We have used flow cytometry to determine expression levels in a panel of human colorectal and other tumour cell lines as well as primary fibroblasts, endothelial and peripheral blood mononuclear cells. Expression was not detected in normal human cells but extensive variation in surface expression of CDCP1 was noted among colorectal and gastric cell lines. In addition, very high levels of expression were found in cervical carcinoma cell lines. These results were confirmed by real-time RT-PCR and Western blotting. We are extending these expression studies to include other primary cell types and staged colorectal tumour samples. However the high expression levels on cells derived from several tumour cell types suggest that CDCP1 may represent a novel target for T cell-mediated immunotherapy. The variations noted in CDCP1 expression between different colorectal cell lines highlight the importance of targeting particular immunotherapeutic strategies to selected groups of patients.
CDCP1 that may be derived from two differentially-spliced transcripts. Furthermore there is evidence that CDCP1 can be cleaved into at least two.

© 2004 Cancer Research UK

### P135

### BFGF-INDUCED APOPTOSIS OF ESFT CELLS IS EFFECTED THROUGH SUSTAINED ACTIVATION OF P38$^{MAPK}$ AND UP-REGULATION OF P75$^{NTR}$

AJK Williamson , JR Boyne , BC Dibling , SA Burchill
Cancer Research UK, St. James's Hospital, Leeds, United Kingdom

In this study the potential role of the mitogen activated protein kinases's c-Jun N-terminal kinase (JNK), extracellular-regulated kinase (ERK) and p38$^{MAPK}$ in bFGF-induced cell death of the Ewing's sarcoma family of tumours (ESFT) has been investigated. The ESFT cell lines TC-32, TTC-466, A673 and the neuroblastoma cell line SK-N-SH were studied. Viable cell number was quantified using the trypan blue exclusion assay and apoptosis by fluorescent associated cell sorting (FACS) of annexin V/PI labelled cells. Total and phosphorylated JNK, ERK, p38$^{MAPK}$ and p75$^{NTR}$ were analysed by ELISA and Western blot. bFGF induced cell death in a time dependent manner in TC-32 and TTC-466, but not in A673 and SK-N-SH cells. Exposure of TC-32 and TTC-466 cells to bFGF induced rapid and sustained phosphorylation of ERK and p38$^{MAPK}$; phosphorylation was detected five minutes after addition of bFGF and was sustained for up to 2 h. Pre-incubation with PD98059 (10μM) inhibited ERK activation and rescued approximately 30% of TC-32 cells from bFGF-induced cell death. However inhibition of p38$^{MAPK}$ signalling with SB202190 (20 μM) rescued 100% of TC-32 cells from bFGF-induced cell death. In contrast ERK and p38$^{MAPK}$ were transiently activated in TC-32 cells treated with the stem cell factor, and in SK-N-SH cells in which bFGF-induced proliferation. However in A673 cells that do not die in response to bFGF p38$^{MAPK}$ was transiently activated, whereas ERK activation was sustained. JNK was transiently activated in all cells, independent of their response to bFGF. Induction of cell death in ESFT cells after exposure to bFGF was associated with an up-regulation of p75$^{NTR}$ expression; knock out of p75$^{NTR}$ with RNAi rescued cells from bFGF-induced cell death. Inhibition of p38$^{MAPK}$ signalling with SB202190 (20μM) prevented up-regulation of p75$^{NTR}$. Induction of cell death by bFGF appears to be mediated by p38$^{MAPK}$-dependent up-regulation of p75$^{NTR}$.

### P136

### EXPRESSION AND EXPLOITATION OF NEURAL-SPECIFIC STATHMINS IN LUNG CANCER

F Aladin [1], E Bennett [2], G Sunderland [1], N Mori [4], N Blake [3], J Coulson[1]
[1] Physiological Laboratory, University of Liverpool, Liverpool, United Kingdom, [2] Human Anatomy and Cell Biology, University of Liverpool, Liverpool, United Kingdom, [3] Medical Microbiology, University of Liverpool, Liverpool, United Kingdom, [4] National Institute for Longevity Sciences, Aichi, Japan

Stathmin proteins destabilise microtubules during cell division or neurite outgrowth. Stathmin 1 is ubiquitous, whereas Stathmin 2 (SCG10), Stathmin 3 (SCLIP) and Stathmin 4 (RB3) exhibit degrees of neural-specificity. SCG10 expression is repressed by the neuron restrictive silencer factor (NRSF) in non-neuronal cells through association with co-factors including histone deacetylase (HDAC) and MeCP2. Small cell lung cancer (SCLC) is characterised by expression of neuroendocrine and neural-restricted genes, and we have described an isoform of NRSF that dysregulates transcriptional repression in SCLC. We now report that stathmin 1 and the neural-specific stathmins are highly expressed in neuroendocrine SCLC. In particular, SCG10 was detected in 8/8 SCLC, 0/4 non-SCLC (NSCLC) and 0/2 normal bronchial epithelial cell lines by RT-PCR. The SCG10 protein was also seen in SCLC cell lines by western blotting and immunocytochemistry. A heterologous SCG10 reporter construct demonstrated highly differential expression between SCLC and NSCLC lines. SCLC-specific expression was repressed by overexpression of NRSF whereas, in NSCLC, the HDAC inhibitor trichostatin A (TSA) de-repressed expression. Transcription of stathmin 1, RB3 and SCLIP is less well characterised, but may also be regulated by NRSF. We therefore investigated the role of HDAC and methylation in lung cells using TSA and 5'-aza-cytidine (5AzaC). In untreated NSCLC, the expression of endogenous stathmins was low or undetectable by RT-PCR, but was increased by treatment with TSA or 5AzaC. As the SCG10 transcript was highly selective for SCLC, this may represent a tumour-specific promoter for viral-directed molecular therapy. Infection of various cell types with adenoviral vectors showed that expression of EGFP dependent on the SCG10 promoter was specific to neuroendocrine tumour cells and was actively restricted in other cell lines by HDAC. Our data suggest the neural-specific stathmins play a role in SCLC and may be exploited in detection or treatment of this disease.

### P137

### THE MORPHOGENIC PROPERTIES OF OLIGOMERIC COLLAGEN XVIII-DERIVED ENDOSTATIN ARE DEPENDENT ON CELL SURFACE GLYCOSAMINOGLYCANS

AR Clamp [1], FH Blackhall [1], A Henrioud [1], GC Jayson [1], K Javaherian[2], JT Gallagher [1], CLR Merry [1]
[1] Cancer Research UK and University of Manchester Dept of Medical Oncology, Christie Hospital NHS Trust, Manchester, United Kingdom, [2] Department of Surgery, Children's Hospital, Harvard Medical School, Boston, United States

Monomeric endostatin (EM) is well-known for its anti-angiogenic properties. In contrast, oligomeric endostatin generated from the intact C-terminal domain of collagen XVIII (ED) induces a promigratory phenotype in endothelial cells which is inhibited by its monomeric counterpart (Kuo et al 2001 J Cell Biol 152:1233). Many pro- and anti-angiogenic growth factors require cell surface glycosaminoglycans (GAG) for their bioactivity and although endostatin is heparin-binding, the role of GAGs in regulating the activity of EM/ED is unclear. In this study we have shown that exogenous glycosaminoglycans can substitute for EM and inhibit the promigratory action of ED in a Matrigel angiogenesis assay. This property is size-dependent as heparin-derived oligosaccharides containing more than 20 monosaccharide residues were optimal inhibitors.

ED was also shown to induce marked morphological changes in Chinese Hamster Ovary cells (CHO)- an epithelial cell line, plated on Matrigel. This novel observation allowed use of a panel of CHO mutants with defined GAG biosynthetic defects. The morphogenic properties of ED were demonstrated to be absolutely dependent on the presence of cell- surface GAG, with heparan sulphate necessary for the wild-type phenotype. Heparan sulphate 2-O-sulphation is not required as CHO pgsF-17 which lack 2-O-sulphotransferase activity respond normally to ED stimulation.

The use of zero-length cross-linking techniques (Grabarek and Gergely 1990 Anal Biochem 185:131) showed that multiple EM molecules can bind to a single oligosaccharide chain simultaneously. This indicates that one potential anti-angiogenic mechanism of EM is to block the GAG-binding sites of ED. This study demonstrates the importance of cell surface GAG binding for the activity of ED and suggests that the activity of ED/EM may not be specific to endothelial cells.

### P138

### CYTOKINE MEDIATED MODULATION OF THE MYC NETWORK IN BARRETT'S METAPLASIA

J. Boult [1], L.S. Young [2], M.J. Campbell [1], C. Tselepis [1]
[1] Division of Medical Sciences, University of Birmingham, Birmingham, United Kingdom, [2] Division of Cancer Studies, University of Birmingham, Birmingham, United Kingdom

Barrett's Metaplasia (BM) is a pre-malignant lesion predisposing to oesophageal adenocarcinoma. Progression of BM to adenocarcinoma is characterised by sequential molecular and genetic events, driven partly by components of the gastro-oesophageal refluxate. Supporting this hypothesis, it has been demonstrated that bile acids and cytokines can induce c-myc expression, consistent with the reported over-expression of this oncogene during the malignant progression of BM. However, the ability of c-myc to act as a transcription factor and to influence cell fate is partly governed by levels of the related Mad family proteins, including Mad-1 and Mxi-1. This study aims to characterise the levels of c-myc, Mad-1 and Mxi-1 in reflux oesophagitis, BM and oesophageal adenocarcinoma. In addition the oesophageal squamous cell line OE21 was used to investigate the effects of the cytokines IL-8, IL-1β and IFN-γ on these proteins.

Using Real Time PCR, we demonstrated a significant induction of c-myc in 5/8 and 5/9 samples of reflux oesophagitis and BM, respectively. In these c-myc positive samples, there was a parallel induction in Mxi-1. In all the samples which failed to show an elevation in c-myc or Mxi-1 there was instead a dramatic induction in Mad-1. Immunohistochemical staining demonstrated that c-myc was localised to the basal layer of the native squamous oesophagus whilst Mxi-1 was expressed in the parabasal layer. Both proteins were localised in areas of intense inflammation in specimens of oesophagitis. Utilising OE21 cells we further demonstrated that IL-8 (10ng/ml), IL-1β (10ng/ml) and IFN-γ (10ng/ml) failed to induce c-myc protein expression over 24 hr. However, Mad-1 protein was suppressed by IL-8 and IL-1β but dramatically induced by IFN–γ. An understanding of the role of cytokines in modulating the Myc-Mad network will provide important insights into factors governing the initiation and malignant progression of BM and may identify targets for therapeutic intervention.

© 2004 Cancer Research UK

### P139

### LOCALIZATION OF P43/ENDOTHELIAL-MONOCYTE-ACTIVATING-POLYPEPTIDE-II (P43/EMAP-II) IN NORMAL AND NEOPLASTIC HUMAN LIVER

W Faisal , P Symonds , JC Murray
Wolfson Digestive Diseases Centre, Queen's Medical Centre, Nottingham University, Nottingham, United Kingdom

**Aim:** First isolated from growth medium conditioned by tumour cells, we have recently shown that p43/EMAP-II induces apoptosis in activated lymphocytes, enabling tumour cells to evade immune response (1). Prolonged immune evasion by hepatitis virus has been linked to hepatocellular carcinogenesis, though the mechanism(s) involved are poorly understood. To investigate p43/EMAP-II as a possible mechanism for virus-induced immunosuppression, we first studied the expression of p43/EMAP-II in normal human liver tissue and normal (THLE-3) and human hepatoma (Alexander cells & HuH-7) cell-line.
**Methods:** Normal liver tissue samples were obtained through autopsy from 3 subjects for immunohistochemistry and analysed using image analysis software. THLE-3 was used for immuno-fluorescence microscopy, while supernatant and cell lysate of all three cell-lines were used for western blot analysis for the detection of EMAP-II.
**Results:** In general, hepatocytes in tissue sections showed strong immunoreactivity for p43/EMAP-II with a punctuate pattern, while the Kupffer cells and bile ductules were generally negative. There is significant gradual increase in expression of the polypeptide from zone-1 to zone-3 of hepatic acini ($p<0.005$). Immunofluorescent microscopy showed positive peri-nuclear granular staining of p43/EMAP-II in THLE-3 cells. Western blot analysis revealed that both normal and neoplastic hepatocytes contain 34kDa precursor form of p43/EMAP-II, which is not secreted in the media under normal culture conditions.
**Conclusion:** The compartmentalized pattern of distribution of p43/EMAP-II in hepatic acini may be an extension of the physiological property of the liver which sequesters more enzymes and cytokines in zone 3 than zone 1, or might be a response to differing levels of oxygen tension in different zones. An assessment of functional significance of strong staining of EMAP-II in liver and factors regulating its expression is currently underway.
Reference: (1) Murray *et al*. (2004). *J Immunol* **172**:274.

### P140

### P43/EMAP II: ISOLATION AND CHARACTERISATION OF PUTATIVE RECEPTOR(S)

S Panjwani , YM Heng , JC Murray
Wolfson Digestive Diseases Centre, Queen's Medical Centre, Nottingham University, Nottingham, United Kingdom

P43/EMAP II, a 22 kDa polypeptide, was first purified from the supernatant of Murine meth A-induced fibrosarcoma cells. This single-chain leaderless polypeptide is initially synthesised as 34 kDa intracellular precursor, associated with the multi-tRNA synthetase complex facilitating aminoacylation activity. The 34 kDa precursor molecule is proteolytically cleaved to release the 22 kDa mature polypeptide extracellularly, by an as yet unidentified pathway, in a similar manner to the leaderless precursor of IL-1β. The secreted form of EMAP II acts as a pleotrophic cytokine with plethora of effects both, *in vitro* and *in vivo* on a variety of cell types. It induces tissue factors on the surface of endothelial cells, increasing the expression of E- and P- selectins and TNFR1, and attracting monocytes and neutrophils *in vitro*. *In vivo*, it sensitises resistant immunological tumours to the anti-tumour effects of TNF. It also induces apoptosis in a number of cell types, including endothelial cells, implying it's pro-inflammatory and anti-angiogenic effects. According to our recent findings, EMAP II inhibits DNA synthesis and cell division, and induces apoptosis in mitogen-activated lymphocytes in PBMC preparations, and in Jurkat cells T-cells, suggesting a novel role in immune evasion by eliminating T-lymphocytes and protecting the tumour cells (1). To facilitate a better understanding and significance of the biological role of EMAP II and its use in cytokine based tumour treatment, we have cloned and purified biologically active mature EMAP II with a C-terminal poly-Histidine-tag to fish out cell-surface receptor(s) from Jurkat cell and PBMC using Ni-NTA affinity chromatography. Our preliminary studies using Jurkat cell biotinylated membrane preparation show co-localisation of EMAP II with biotinylated membrane proteins suggesting possible receptors for EMAP II. We intend to identify and characterise the co-localised proteins and study their distribution on cell surface and cell types.
(1) Murray *et al*. (2004). *J Immunol* **172**:274.

### P141

### STRUCTURE AND REGULATION OF THE ENDOTHELIAL MONOCYTE ACTIVATING POLYPEPTIDE II (EMAP-II) GENE

YM Heng , J Edgson , P Symonds , JC Murray
Wolfson Digestive Diseases Centre, Queen's Medical Centre, Nottingham University, Nottingham, United Kingdom

The 34kDa Endothelial Monocyte Activating Polypeptide II (EMAP-II), first found in tumor cell supernatants, is believed to be identical to the p43 auxiliary component of the mammalian aminoacyl multisynthetase complex (1). We recently showed that in the tumor environment this protein is a highly expressed extracellular molecule that inhibits proliferation and induces apoptosis in lymphocytes (2). This may constitute a novel immunosuppressive pathway in certain tumors. **Aim**: We are attempting to understand the regulation of EMAP-II expression by studying the structure and regulatory regions of the gene, as well as the tissue distribution of its expression. **Methods**: We searched genome databases, performed 5' RACE to map RNA start sites, Northern hybridisations to determine RNA distribution and size, and Western blots to determine protein size in hamster and human cell extracts. **Results**: The human EMAP-II gene spans ~30kb and 7 exons, and is located in a head-to-head orientation with a novel gene HSPC302 on chromosome 4q22-25. The intergenic region flanked by the 2 genes showed strong bidirectional promoter activity in reporter asays. By Northern analysis, EMAP-II mRNA was relatively weakly expressed in most normal tissues examined, but most highly expressed in normal human testis, as 3 different transcripts. All tumour cells examined also exhibit 3 mRNA species, albeit in varying ratios. Using Western blotting techniques, we detected EMAP-II as a predominant 34kDa protein in human and hamster cells. However, hamster appears to also express a minor 43kD form, presumably due to 'leaky' translation from an upstream in-phase ATG codon. This minor form was not detected in human and its significance remains unclear. **Conclusion:** We conclude that the 34kD EMAP-II form is highly conserved in mammals, and in addition to its central role in protein translation in all cells, may 'moonlight' as an immunosuppressive molecule under certain tumour conditions.
Reference:
(1) Quevillon et al., (1997). *J. Biol. Chem.* **272** (51) : 32573-9
(2) Murray *et al*. (2004). *J Immunol* **172**:274.

### P142

### ACTIVATION OF FIBROBLAST GROWTH FACTOR 8 (FGF8) EXPRESSION BY TRICHOSTATIN A: A POTENTIAL ROLE FOR PI3 KINASE

K Armstrong , V.J. Gnanapragasam , K Halkidou , C.N. Robson , H.Y. Leung
Northern Institute for Cancer Research, University of Newcastle upon Tyne, Newcastle upon Tyne, United Kingdom

FGF8 expression is tightly controlled in embryogenesis and carcinogenesis [1]. As histone acetyltransferases (HATs) and histone deacetylases (HDACs) critically regulate gene expression [2], we tested the role of acetylation in the transcriptional control of FGF8 expression by challenging human prostate cancer cells with trichostatin A (TSA), a reversible HDAC inhibitor.
Transfecting a human *fgf8* promoter-luciferase reporter construct into PC3M prostate cancer cells, we observed a 4-6 fold induction of promoter function by TSA (200nM) treatment. Endogenous FGF8 expression in LNCaP and PC3M cells was up-regulated in a dose dependent manner by TSA treatment, with a maximal effect observed at 200nM. Promoter deletion reporter assays revealed a region 996-1613bp upstream of the transcriptional start site to be required for TSA induced promoter activity. PI3 kinase has been implicated as a mediator of HDAC inhibitor function [3]. TSA induced *fgf8* promoter reporter function and endogenous FGF8 expression were abolished by specific inhibition of PI3 kinase with LY294002 (25μM). WST-1 assays showed reduced proliferation with increasing TSA concentrations, and FACS analysis confirmed cell cycle arrests at G1/0 and G2/M in prostate cells (LNCaP, DU145 and PC3M) treated with TSA at 20nM and 2μM respectively. However, using a specific neutralising antibody against FGF8, we excluded FGF8 as a mediator in the suppression of proliferation. Our ongoing work aims to define the functional and *in vivo* significance of TSA induced FGF8 expression.
***References***: [1] Leung, H. Y., C. Dickson, C. N. Robson and D. E. Neal (1996) Oncogene 12(8) 1833. [2] Timmermann, S., H. Lehrmann, A. Polesskaya and A. Harel-Bellan (2001) Cell Mol Life Sci 58(5-6) 728. [3] Kim, Y. K., J. W.Han, Y. N. Woo, J. K. Chun, J. Y. Yoo E. J. Cho, S. Hong, H. Y. Lee, Y. W. Lee, and H. W. Lee (2003) Oncogene 22(38) 6023.

© 2004 Cancer Research UK

### P143

### ACQUISITION OF ENDOCRINE RESISTANCE: CHANGES IN TRANSCRIPTION COMPLEX ASSEMBLY IN RESISTANT HUMAN BREAST CANCER CELL LINES.

B Kuske , K MacLeod , C Naughton , K Moore , A O'Donnell , R Clarke , W Miller , S Langdon , D Cameron
Cancer Research Centre, University of Edinburgh, Edinburgh, United Kingdom

Endocrine therapy is a major treatment modality for breast cancer. However, despite initial responsiveness to anti-oestrogens, most tumours will eventually recur with acquired anti-oestrogen resistance. Knowledge on the pathways responsible for the development of endocrine resistance remains limited. Oestrogen activation involves oestrogen receptor binding to DNA with subsequent cyclic assembly of a transcription complex at specific promoters incorporating cofactors such as the p160 family (Cell, Vol 103, 843,2000). As part of a study designed to identify potential resistance mechanisms, we have investigated oestrogen receptor (ER) mediated transcription activation at the pS2 promoter in resistant variants of the MCF-7 cell line.
Oestrogen sensitive MCF-7 human breast cancer cell were compared with variant LCC1, LCC2, LCC9 and LY2 lines which exhibit relative degrees of oestrogen insensitivity. Using quantitative RT-PCR, we observed that addition of 1nM $E_2$ for 48h increased pS2 mRNA by 100-fold in MCF-7 cells, but only 2-4 fold in all 4 variant cells where expression in the absence of $E_2$ was constitutively high. These results broadly reflect the growth response to $E_2$ in these lines. Expression levels of a number of ER coregulators (AIB, SRC-2, SRC-1, REA, NCoR, SMRT) were determined by RT-PCR and Western blot. Several coregulators showed differential expression.Using chromatin immuno-precipitation we identified increasing H4 acetylation (indicating active transcription), recruitment of ERα and the cyclic recruitment of AIB at the pS2 promoter in MCF-7 cells on addition of $E_2$. In contrast, in LCC1 cells, we detected constant H4 acetylation and recruitment of ERα and AIB on $E_2$ addition. Interestingly, LCC9 cells show active constitutive H4 acetylation but no recruitment of ERα or AIB on $E_2$ addition. These results reveal considerable differences in the assembly of a transcription complex in ER mediated gene transcription at the pS2 promoter and may help identify oestrogen-independent mechanisms.

### P144

### MODIFED SER[118] PHOSPHORYLATION OF OESTROGEN RECEPTOR MAY CONTRIBUTE TO ENDOCRINE RESISTANCE IN BREAST CANCER CELLS

K Moore , C Naughton , B Kuske , K MacLeod , R Clarke , J Smyth , W Miller , D Cameron , S Langdon
Cancer Research Centre, University of Edinburgh, Edinburgh, United Kingdom

Changes in oestrogen receptor (ER) activation may cause endocrine insensitivity and resistance in breast cancer (BC) by ligand-independent stimulation of ERα phosphorylation of serine residues in the AF-1 region of the receptor. Such phosphorylations contribute to ER activation ( by enhancing cofactor binding) and can be mediated via Erk and Akt which phosphorylate Ser[118] and Ser[167] respectively. These processes are stimulated by growth factors and may be most relevant in tumours where the EGF receptor or erbB2 are overexpressed.
We are currently examining these pathways in a series of endocrine-resistant MCF-7 resistant cell lines (LCC1, LCC2 and LCC9) which are relatively insensitive to both $E_2$ and anti-oestrogens (in contrast to wild type MCF-7). Changes in ER phosphorylation have been evaluated by Western blotting using phospho-specific antibodies after exposure to $E_2$ (1nM) and tamoxifen (TAM; 1μM) in culture. While $E_2$ markedly stimulates Ser[118] phosphorylation in the MCF-7 wild type line (at least 5-fold), phosphorylation remains at a constantly low level in LCC9 cells. TAM also stimulates phosphorylation in the wild type MCF-7 line when added in the absence of $E_2$ but reduces the level of phosphorylation produced by $E_2$ when both are present together. In the LCC1 and LCC2 lines, the $E_2$-activated level of Ser[118] phosphorylation is not fully reduced by TAM. TGFα (1nM) also stimulates Ser[118] phosphorylation but with a band shift pattern different to that produced by $E_2$ or TAM**.** These mechanisms are currently being investigated in primary breast cancers by relating phosphorylation events to DNA binding and downstream transcription of $E_2$-regulated genes. These results suggest that in endocrine-resistant cells, ER-α might be poorly phosphorylated by $E_2$ and/or TAM may be unable to reverse fully the $E_2$-activated phosphorylation.

### P145

### MFISH IDENTIFIES KARYOTYPIC VARIATION BETWEEN INDEPENDENTLY CULTURED STRAINS OF THE MDA-MB231 BREAST CANCER CELL LINE

MB Watson , MJ Lind , PJ Drew , L Cawkwell
PGMI, University of Hull, HULL, United Kingdom

Established cell lines derived from breast cancers provide important models, which can be used to study the genetics, biochemistry and dynamics of breast cancer in vitro. However, the very nature of these cell lines, along with their widespread and prolonged culture in plastic, may eventually lead to genotypic and phenotypic variations between strains cultured by different research groups. The aim of this study was to investigate the in vitro genetic divergence at the chromosome level of the breast cancer cell line MDA-MB231.
Multicolour fluorescence in situ hybridisation (MFISH) allows rapid detection, discrimination and karyotyping of all chromosomes within a single metaphase spread. Strains of MDA-MB231 were obtained from 3 different laboratories (Hull, York & USA) and subjected to MFISH analysis.
As expected, many Karyotypic aberrations were identified, which were common to all strains of this cell line. For example, the marker chromosomes derivative(2)t(2;12;8) and der(2)t(8;2). However, several other unique abnormalities were identified between strains enabling the production of a map of Karyotypic divergence. For example der(6)t(6;19;12;8), which was unique to the hull strain and der(19)t(19;X;5), which was unique the USA strain. This study identifies the need for the potential genetic divergence of cell lines cultured extensively in the laboratory to be taken into account during the interpretation of experimental data.

### P146

### HYPOXIA-REGULATED GENE EXPRESSION IN RHABDOMYOSARCOMA CELL LINES

N Hermitage [1], A Evans [1], P Houghton [2], I Stratford [3], E Estlin [4], R Airley [1]
[1] Liverpool John Moores University, Liverpool, United Kingdom,
[2] St Judes Childrens Research hospital, Memphis, United States,
[3] University of Manchester, Manchester, United Kingdom, [4] Royal Manchester Childrens Hospital, Manchester, United Kingdom

Rhabdomyosarcoma, the most common type of paediatric soft tissue sarcoma, is denoted by high malignancy, a thirty percent mortality rate, with peak incidence in 1-4 year olds. Hypoxia is frequently observed in solid tumours within areas of rapid cell proliferation served by quantitatively and/or qualitatively poor vascularity. To increase intracellular nutrients during hypoxic stress, malignant cells adapt through up-regulation of hypoxia-inducible genes such as the facilitative glucose transporter-1 Glut-1. Tumours with a high hypoxic fraction have less favourable prognosis and poor treatment response. This has been demonstrated for cancers of the head and neck, and cervix. Glut-1 is over expressed and predicts poor prognosis in a wide range of tumours, and has been clinically validated as an intrinsic marker of hypoxia. In paediatric oncology intense drug and radiation therapy has to be balanced against the risks of late sequelae such as growth and development retardation. Rational application of aggressive treatment to hypoxic areas of the tumour may be one way of overcoming this therapeutic problem. We have recently found that Glut-1 and Glut-3 are expressed peri-necrotically in 10/20 and 7/14 rhabdomyosarcoma samples respectively. To complement these findings, we have assessed the expression of hypoxia-medicated genes Glut-1, Glut-3 and VEGF in rhabdomyosarcoma cell lines. Immunohistochemistry was carried out on formalin-fixed, paraffin-embedded pellets of Rh30 and Rh36 cell lines following 18 hours in anoxic conditions. In both cell lines, there was no constitutive Glut-1 expression but after exposure to anoxia moderate to heavy Glut-1 expression was apparent. Glut-3 was detected in RH36 cells at equal levels in normoxic and anoxic samples, but there was no Glut-3 detected in RH30 cells. Therefore, the Glut-1 seen in biopsy samples may reflect the existence of hypoxia in the tumour, but other factors may be regulating tumour Glut-3.

© 2004 Cancer Research UK

### P147

### AN INDUCIBLE SYSTEM OF GENE EXPRESSION PROVIDES THE MEANS TO INVESTIGATE THE FUNCTIONS OF OPCML

E. Ntougkos [1], G.J. Rabiasz [1], E.P. Miller [1], H. Gabra [2], J.F. Smyth [1], G.C. Sellar [1]
[1] Cancer Research UK, Edinburgh, United Kingdom, [2] Imperial College, London, United Kingdom

*OPCML*, alternatively known as *OBCAM* (*O*pioid *B*inding *C*ell *A*dhesion *M*olecule), encodes a member of the IgLON subfamily of cell adhesion molecules. *OPCML* is frequently inactivated in epithelial ovarian cancer (EOC) and exhibits properties of a tumour suppressor gene, such as growth suppression *in vivo* and *in vitro* and enhanced cell-cell aggregation *in vitro*. It is the first IgLON reported to be involved in cancer.
We have employed an inducible system of gene expression to investigate the basic biology of OPCML. The BD Tet-On™ system allows tightly regulated expression after induction with doxycycline, while avoiding obstacles of clonal variation and over-expression artefacts. Two *OPCML* inserts have been expressed in HeLa Tet-On™ cells: a wild-type *OPCML* coding sequence and one harbouring a loss-of-function mutation that occurs in EOC. The wild-type and mutant clones exhibit dose-dependent *OPCML* expression. Tight regulation of *OPCML* expression, with minimal leaky expression, is evident both at the RNA level, according to real-time RT-PCR data, and at the protein level, according to western blotting. RNA and protein data are well correlated.
We are currently using this platform of investigation, and assays that examine cellular properties, such as cell growth and attachment, in order to unravel the functions of OPCML. In addition to the pursuit of a cellular phenotype in the context of HeLa cells, we are also interested in the importance of the level of *OPCML* expression in effecting this phenotype. Preliminary evidence suggests that although induction of OPCML does not result in overt growth suppression, it may account for an increased attachment to extracellular matrix components, such as fibronectin. The significance of a cell attachment-related phenotype in HeLa cells will be validated further.

### P148

### EXPRESSION OF CHEMOKINES IL-8 AND GCP-2 AND THEIR RECEPTORS CXCR1 AND CXCR2 IN SMALL AND NON-SMALL CELL LUNG CANCER CELL LINES

YM Zhu , D Flower , PJ Woll
University of Sheffield, Sheffield, United Kingdom

Interleukin (IL)-8 and Granulocyte chemotactic protein (GCP)-2 are potent chemokines and angiogenic factors that both act through the CXCR1 and CXCR2 receptors. Here, we studied the expression of IL-8, GCP-2, CXCR1 and CXCR2 in 3 non-small cell lung cancer (NSCLC) cell lines and 5 small cell lung cancer (SCLC) cell lines.
**Results:** IL-8 and GCP-2 proteins were measured by ELISA in conditioned medium (CM). NSCLC cells produced high levels of IL-8 at 24 hours (196, 6153 & 584 pg/ml/$10^6$ of A549, H460 & MOR/P, respectively) and at 48 hours (1227, 134615 & 7299 pg/ml) whereas IL-8 was very low or absent in CM of SCLC. In contrast, SCLC cells produced high levels of GCP-2 at 24 hours (390, 1935, 38 & 125 pg/ml/$10^6$ of H345, H711, GLC-19 & Lu165, respectively) and at 48 hours (781, 3225, 128 & 125 pg/ml), whereas NSCLC did not produce GCP-2. We studied the expression of CXCR1 and CXCR2 by FACS in these cell lines. In NSCLC cell lines, 32-47% of cells expressed CXCR1 and 9-28% expressed CXCR2. In SCLC cell lines, 28-56% of cells expressed CXCR1 and 8-38% expressed CXCR2. Expression of CXCR1 and CXCR2 mRNA was also assessed by RT-PCR and the results were in agreement with FACS analysis. To investigate the potential mitogenic role of IL-8 in lung cancer, we treated IL-8 producing NSCLC cells with neutralising anti-IL-8 antibody and non-IL-8 producing SCLC cells with exogenous IL-8. Cell proliferation was determined by MTT assay. NSCLC cell proliferation was significantly inhibited by anti-IL-8 antibody in a dose-dependent fashion. The SCLC cells were stimulated by rIL-8 in a dose- and time-dependent fashion.
**Conclusion:** Our results show that SCLC and NSCLC cells express CXCR1 and CXCR2 receptors. We have found significant differences in ligand expression, as NSCLC cells produce IL-8 whereas SCLC cells produce GCP-2. IL-8 is mitogenic for lung cancer cells. CXC chemokines may therefore be a novel target for lung cancer treatment.

### P149

### THE INDUCTION AND MAINTENANCE OF SENESCENCE IN HCT116 CELLS BY DNA DAMAGING AGENTS

A.O Oke , S.P Joel
Department of Medical Oncology, St Bartholomew's Hospital, West Smithfield, London, United Kingdom

Senescence is a major tumour suppressor mechanism limiting the proliferative capacity of cells in vivo, and is a major barrier to tumorigenesis. We have previously showed that the topoisomeraese-I inhibitor SN-38 (the active metabolite of irinotecan) is able to induce senescence in tumour cells expressing wild-type p53, and that p21 plays a central role in the onset of senescence, which is then maintained by p16 (te Poele et al, Can Res 62, 1876–1883, 2002). We have investigated this further using the HCT116 colorectal cell line panel (including $p53^{wt}$, $p53^{-/-}$ and $p21^{-/-}$ cells), which have a hypermethylated p16 promoter.
SN-38 induced G2 arrest was seen in all cells, but subsequent cell death was more extensive in $p53^{-/-}$ and $p21^{-/-}$ cells than in $p53^{wt}$ cells that remained viable but arrested and stained +ve for β-galactosidase (β-gal) activity. At high drug concentration (100 ng/ml) surviving $p53^{-/-}$ and $p21^{-/-}$ cells, although low in number, also stained +ve for β-gal, but lost this activity and resumed growth after 8 days in drug-free medium. In contrast, $p53^{wt}$ cells did not lose β-gal staining nor resumed growth until 22 days drug-free, confirming the importance of p16 in maintaining senescence. Additionally, $p53^{wt}$ cells in which senescence had been induced by exposure to SN-38 showed decreased sensitivity to taxol, 5-FU and cisplatin compared to control cells using the sulphorhodamine blue assay.
These data confirm the importance of p53 and p21 in the induction of senescence. Failure to maintain senescence in HCT116 $p53^{wt}$ cells is likely to be due to silencing of the p16 gene by hypermethylation. The decreased sensitivity of senescent cells to subsequent cytotoxic drug exposure may have clinical significance. We are currently investigating pathways involved in the induction and loss of the senescent phenotype in these cells using expression microarrays.

### P150

### REGULATION OF CXCR1 AND CXCR2 RECEPTORS IN NSCLC CELL LINES

S Webster , YM Zhu , PJ Woll
University of Sheffield, Sheffield, United Kingdom

Interleukin-8 (IL-8) is a chemokine with angiogenic and mitogenic effects in cancer. Expression of IL-8 in non-small cell lung cancer (NSCLC) is associated with angiogenesis, metastasis, and poor patient outcome. We have identified IL-8 as a putative autocrine growth factor for NSCLC. Here, we investigated the cell surface expression of CXCR1 and CXCR2 by flow cytometry in 3 NSCLC cell lines, and regulation of receptor expression by cell density and endogenous IL-8.
**Results:** Cell membrane expression of CXCR1 and CXCR2 was detected by flow cytometry. CXCR1 were more commonly expressed than CXCR2. Interestingly, higher numbers of both receptors were detected when cells were removed from the flasks by scraping than by trypsinisation: 45% vs 12% for CXCR1 in H460 cells, 40% vs 14% in MOR/P cells and 32% vs 4% in A549 cells. For CXCR2, 20% vs 9% in H460 cells, 9% vs 5% in MOR/P and 13% vs 3% in A549 cells. We further analysed cell membrane CXCR1 and CXCR2 in 50% and 100% confluent cells. We found that totally confluent cells expressed much lower levels of CXCR1 and CXCR2 than 50% confluent. The CXCR1 positive cells were downregulated from 43% to 22% in H460 and 25% to 15% in MOR/P. The CXCR2 positive cells were downregulated from 28% to 6% in H460 and 9% to 6% in MOR/P. H460 and MOR/P cells constitutively produced IL-8. IL-8 concentration measured by ELISA was 65ng/ml in H460 and 3.5 ng/ml in MOR/P when cells were confluent at 48h. IL-8 concentration was halved in H460 and in MOR/P when cells were 50% confluent. These results suggest that CXCR1 and CXCR2 might be internalised at higher concentrations of IL-8.
**Conclusions:** Our results suggest that CXCR1 and CXCR2 are commonly expressed on NSCLC cell membranes and their expression is affected by cell density. Downregulation of membrane CXCR1 and CXCR2 (internalization) in high density cells may be affected by higher level of endogenous IL-8 in the medium.

© 2004 Cancer Research UK

